# Genomic Profiling of Submucosal-Invasive Gastric Cancer by Array-Based Comparative Genomic Hybridization

**DOI:** 10.1371/journal.pone.0022313

**Published:** 2011-07-21

**Authors:** Akiko Kuroda, Yoshiyuki Tsukamoto, Lam Tung Nguyen, Tsuyoshi Noguchi, Ichiro Takeuchi, Masahiro Uchida, Tomohisa Uchida, Naoki Hijiya, Chisato Nakada, Tadayoshi Okimoto, Masaaki Kodama, Kazunari Murakami, Keiko Matsuura, Masao Seto, Hisao Ito, Toshio Fujioka, Masatsugu Moriyama

**Affiliations:** 1 Department of Molecular Pathology, Faculty of Medicine, Oita University, Oita, Japan; 2 Department of General Medicine, Faculty of Medicine, Oita University, Oita, Japan; 3 Department of Gastrointestinal Surgery, Faculty of Medicine, Oita University, Oita, Japan; 4 Department of Computer Science/Scientific and Engineering Simulation, Nagoya Institute of Technology, Nagoya, Japan; 5 Department of Gastroenterology, Faculty of Medicine, Oita University, Oita, Japan; 6 Division of Molecular Medicine, Aichi Cancer Center Research Institute, Nagoya, Japan; 7 Division of Organ Pathology, Department of Microbiology and Pathology, Faculty of Medicine, Tottori University, Yonago, Japan; Tor Vergata University of Rome, Italy

## Abstract

Genomic copy number aberrations (CNAs) in gastric cancer have already been extensively characterized by array comparative genomic hybridization (array CGH) analysis. However, involvement of genomic CNAs in the process of submucosal invasion and lymph node metastasis in early gastric cancer is still poorly understood. In this study, to address this issue, we collected a total of 59 tumor samples from 27 patients with submucosal-invasive gastric cancers (SMGC), analyzed their genomic profiles by array CGH, and compared them between paired samples of mucosal (MU) and submucosal (SM) invasion (23 pairs), and SM invasion and lymph node (LN) metastasis (9 pairs). Initially, we hypothesized that acquisition of specific CNA(s) is important for these processes. However, we observed no significant difference in the number of genomic CNAs between paired MU and SM, and between paired SM and LN. Furthermore, we were unable to find any CNAs specifically associated with SM invasion or LN metastasis. Among the 23 cases analyzed, 15 had some similar pattern of genomic profiling between SM and MU. Interestingly, 13 of the 15 cases also showed some differences in genomic profiles. These results suggest that the majority of SMGCs are composed of heterogeneous subpopulations derived from the same clonal origin. Comparison of genomic CNAs between SMGCs with and without LN metastasis revealed that gain of 11q13, 11q14, 11q22, 14q32 and amplification of 17q21 were more frequent in metastatic SMGCs, suggesting that these CNAs are related to LN metastasis of early gastric cancer. In conclusion, our data suggest that generation of genetically distinct subclones, rather than acquisition of specific CNA at MU, is integral to the process of submucosal invasion, and that subclones that acquire gain of 11q13, 11q14, 11q22, 14q32 or amplification of 17q21 are likely to become metastatic.

## Introduction

Gastric cancer remains one of the most deadly diseases, despite its steadily declining trend worldwide. Overall, mortality due to gastric cancer is estimated to be 700,000 cases annually (10.4% of all cancer-related deaths), ranking 2nd only after lung cancer [1]. Clinical outcome is better when the tumor cells are confined to the mucosa. However, once the tumor cells pass through the muscularis mucosa, the clinical outcome becomes worse, since the risk of lymph node metastasis, which is one of the most important prognostic factors in gastric cancer, increases significantly to 18% or more, compared with less than 4% when the tumor cells remain limited to the mucosa [2], [3]. Therefore, a better understanding of the mechanisms involved in the process of submucosal invasion is required.

It is currently recognized that multistep accumulation of genetic abnormalities is responsible for the onset and progression of various cancers [4]. In fact, it has been reported that the total number of genomic aberrations increases with tumor progression in various types of tumors [5]. We also found that the frequencies of gains at 20q, 20p12, 1q42, 3q27 and 13q34 and losses at 4q34-qter, 4p15, 9p21, 16q22, 18q21 and 3p14, which had been frequently detected in gastric cancer, were more frequent in AGC than in EGC [6]. Meanwhile, it has recently been reported that, during the course of tumor progression, a single tumor cell of origin evolves into several genetically distinct subpopulations through the acquisition of a wide variety of genomic aberrations. The resulting tumor mass, which is composed of genetically heterogeneous subpopulations, is considered to become resistant to a variety of environmental selection pressures [7], [8], [9], [10].

Array-based comparative genomic hybridization (array CGH) provides information about genomic copy number aberrations (CNAs) across the entire genome [11]. Moreover, CGH is also applicable to the study of intratumoral genomic heterogeneity [12], [13], [14], [15]. Although several groups have used array CGH to identify regions harboring oncogenic or tumor-suppressive genes in gastric cancer [6], [16], [17], [18], [19], [20], [21], [22], [23], [24], [25], CNAs related to submucosal invasion and the early phase of lymph node metastasis have not yet been determined. Furthermore, since most previous studies of CNAs in gastric cancer have analyzed only one sample for each tumor, details of the heterogeneity of genomic profiles within a single gastric cancer have remained largely unclear.

In this study, we investigated the involvement of genomic CNAs in the process of submucosal invasion and lymph node metastasis in early gastric cancer. For this purpose, we collected tumor samples from different portions of the same tumor separately, analyzed their genomic profiles by array CGH, and compared the genomic profiles between paired samples of mucosal (MU) and submucosal (SM) portions, and SM portion and lymph node (LN) metastasis. Furthermore, by comparing the CNAs between metastatic and non-metastatic submucosal-invasive gastric cancers (SMGC), we identified the candidate CNAs related to LN metastasis of early gastric cancer.

## Materials and Methods

### Ethics Statement

This study was approved by the ethics committee of Oita University Hospital (Approval No P-05-04). Informed written consent was obtained from all patients and/or their families.

### Patients, tissue samples and extraction of genomic DNA

**Figure 1:**
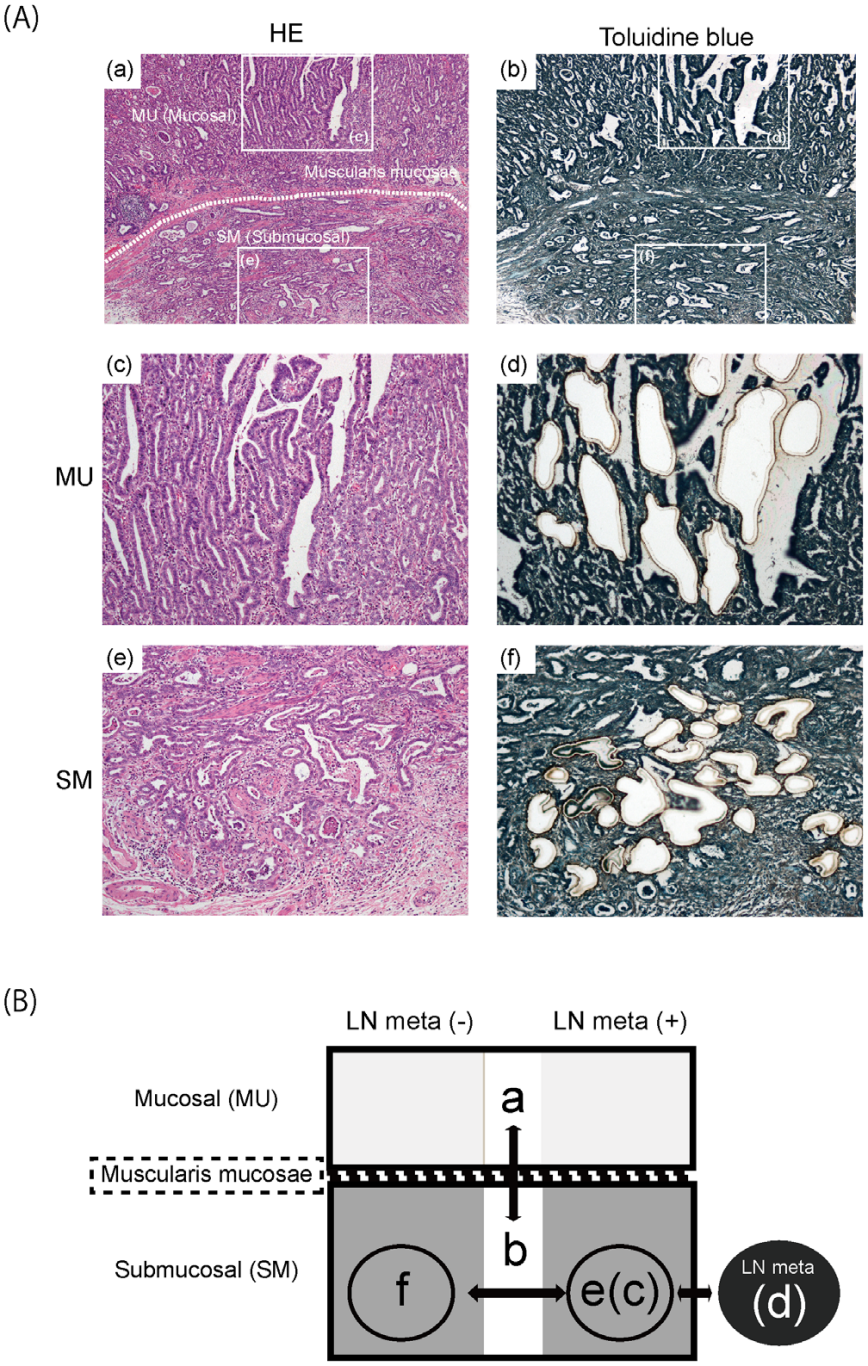
Typical histology of submucosal invasive gastric cancer and experimental design. (A) HE (a, c and e) and toluidine blue (b, d and f) staining of case 18 in low- (a and b) and high- (c, d, e and f) power views. Tissue sections after microdissection are shown in (d) and (f). (B) Overview of the experimental design. First, genomic profiles of 23 MU samples (a) were compared with those of paired 23 SM samples (b). Next, the genomic profiles of 9 SM samples (c) were compared with those of the corresponding paired 9 LN samples (d). Finally, genomic profiles were compared between the SM of 12 cases with LN metastasis (e) and the SM of 15 cases without metastasis (f). The individual samples of (a)–(b) are indicated by superscripts in Table 1.

Twenty seven SMGCs were surgically resected at Oita University Hospital. Tissue sections were cut from formalin-fixed, paraffin-embedded tissue, and stained with hematoxylin-eosin (HE) for histological analysis and with toluidine blue (Wako, Osaka, Japan) for extraction of genomic DNA (Figure 1A). Using laser-capture microdissection, we collected 1 to 3 samples from the MU, SM and/or metastatic LN portion of the same SMGC tissue separately. As a result, we were able to obtain a total of 59 samples from 27 patients (Table 1). All samples included a proportion of tumor cells exceeding 70% of the total. Genomic DNA was extracted in according to the standard proteinase K digestion method, followed by phenol/chloroform extraction. Non-neoplastic gastric tissue from the same patients was used as a normal control.

**Table 1:** Clinicopathological characteristics of patients.

Case	Age	Gender	LN meta	Collected sample histology*	
				Intramucosal	Submucosal
1	77	male	+^d^	pap^a^	mod^b^^,^ c ^,^ e
2	81	female	+^d^	N.A.	por^b^^,^ c ^,^ e
3	70	male	+^d^	por^a^	por^b^^,^ c ^,^ e
4	60	male	+^d^	well^a^	por^b^^,^ c ^,^ e
5	47	female	+ (N.A.)	por^a^	por^b^^,^ e
6	71	male	+^d^	well^a^	mod^b^^,^ c ^,^ e
7	64	male	+^d^	mod^a^	mod^b^^,^ c ^,^ e
8	77	female	+^d^	por^a^	por^b^^,^ c ^,^ e
9	57	male	+^d^	N.A.	mod^c^^,^ e
10	76	female	−	well^a^	pap^b^^,^ f
11	81	male	−	well^a^	por^b^^,^ f
12	77	female	−	well^a^	mod^b^^,^ f
13	79	male	−	well^a^	well^b^^,^ f
14	71	male	−	por^a^	por^b^^,^ f
15	78	male	−	N.A.	por^f^
16	78	female	−	well^a^	well^b^^,^ f
17	79	male	−	por^a^	por^b^^,^ f
18	67	male	−	mod^a^	mod^b^^,^ f
19	81	female	−	mod^a^	mod^b^^,^ f
20	69	female	−	por^a^	por^b^^,^ f
21	76	female	+^d^	mod^a^	well^b^^,^ c ^,^ e
22	67	male	+ (N.A.)	well^a^	well^b^^,^ e
23	70	male	−	well^a^	well^b^^,^ f
24	74	male	−	mod^a^	mod^b^^,^ f
25	76	male	+ (N.A.)	N.A.	pap^e^
26	50	male	−	pap^a^	pap^b^^,^ f
27	70	male	−	mod^a^	mod^b^^,^ f

por = poorly-differentiated adenocarcinoma;

mod = moderately-differentiated adenocarcinoma;

well = well-differentiated adenocarcinoma;

pap = papillary adenocarcinoma.

*Japanese classification of gastric cancer.

**Figure 2:**
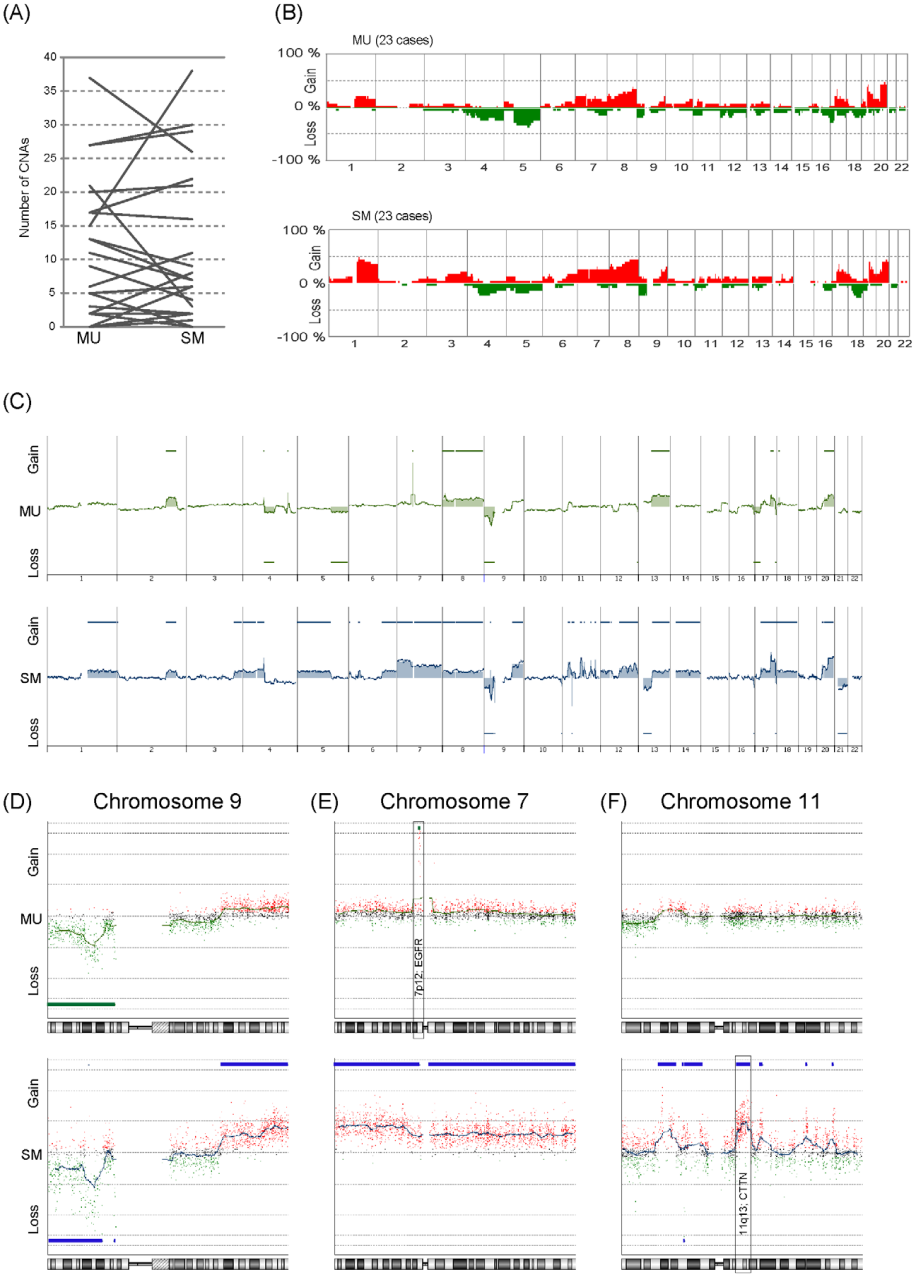
Comparison of CNAs between paired MU and SM portions. (A) Comparison of the number of CNAs in the MU and SM portions. For this analysis, samples indicated by ‘a’ and ‘b’ in Table 1 were used. (B) Genome-wide frequencies of CNAs in MU and the corresponding paired SM in 23 cases. Horizontal lines: oligonucleotide probes are shown in order from chromosomes 1 to 22. Within each chromosome, clones are shown in order from the p telomere to the q telomere. Vertical lines: frequency (%) of gains (positive axis) and losses (negative axis) are shown for each probe. (C–F) Representative genomic profile of MU and SM portions of SMGC. Whole genomic profiles of the paired MU (above) and SM (below) portions from case 4 are shown in (C). Detailed genomic profiles of Chr9, Chr7 and Chr11 are shown in (D), (E) and (F), respectively. Horizontal lines above the center represent regions of gain, and those below the center represent regions of loss. Both MU and SM show similar genomic patterns in chromosome 9p (D). However, amplification of 7p12, where the EGFR gene is located, is detected only in the MU portion (E), and gain of 11q13, where the CTTN gene is located, is detected only in the SM portion (F).

N.A. = Samples that were not analyzed.

a,b Samples that were used for analysis shown in Figure 2(A) and (B).

c,d Samples that were used for analysis shown in Figure 4(A) and (B).

e,f Samples that were used for analysis shown in Figure 5(A).

### Array CGH and data analysis

Array-CGH analysis was performed using 44 K oligonucleotide CGH arrays (Agilent Technologies Inc., Palo Alto, CA). Labeling and hybridization were performed according to the protocol provided by Agilent Technologies Inc. Briefly, 0.85–2 µg of tumor DNA and an equal amount of control DNA were digested with AluI and RsaI (Promega, Madison, WI, USA) for 24 h at 37°C. The digested tumor and the control DNA were labeled with Cy5-dUTP and Cy3-dUTP, respectively, using a Genomic DNA Labeling Kit Plus (Agilent), purified with Microcon YM-30 filters (Millipore, Billerica, MA, USA), and concentrated to 80.5 µl. Equal amounts of tumor and control DNAs were subsequently pooled and mixed with human Cot-1 DNA, dissolved in hybridization buffer (Agilent Oligo aCGH Hybridization Kit; Agilent Technologies), denatured and hybridized to the CGH array at 65°C for 24 h. Glass slides were washed and then scanned in accordance with the manufacturer's instructions.

Microarray images were analyzed using FEATURE EXTRACTION v.9.5.3.1 (Agilent Technologies) with linear normalization (protocol CGH-v4_95_Feb07), and the resulting data were imported into DNA Analytics v.4.0.81 (Agilent Technologies). Following normalization of raw data, the log2ratio of Cy5 (tumor) to Cy3 (Control) was calculated. Aberrant regions were determined by the ADM-2 algorithm at a threshold of 8.0. To detect gains and losses, we set the values of parameters for aberration filters as: minimum number of probes in region 2, minimum absolute average log2ratio for region 0.26, maximum number of aberrant regions 10000, and percentage penetrance per feature 0. Similarly, to detect amplifications and deletions, we set the values of parameters for aberration filters as: minimum number of probes in region 2, minimum absolute average log2ratio for region 1.0, maximum number of aberrant regions 10000, and percentage penetrance per feature 0. Data generated by probes mapped to the X and Y chromosomes were eliminated. Genomic positions of probes and aberrant regions were based on the UCSC March 2006 human reference sequence (hg18) (NCBI build 36 reference sequence). All data are MIAME compliant (http://www.mged.org/Workgroups/MIAME/miame.html) and the raw data have been deposited in the MIAME-compliant GEO database (http://www.ncbi.nlm.nih.gov/geo/, accession number GSE26800). An overview of the experimental design is shown in Figure 1B. For comparison of CNAs between paired MU and SM portions, we selected 23 cases from the total of 27 (Figure 1B, a and b), since the genomic profiles of both portions in these cases had been successfully analyzed. Similarly, for comparison of CNAs between paired SM and LN portions, we selected 9 of the 12 cases with a LN portion (Figure 1B, c and d). Furthermore, we compared the frequencies of CNAs between the cases with and without LN metastasis (Figure 1B, e and f).

### Immunohistochemistry

Immunohistochemistry was performed as described previously [21] using anti-EGFR (1∶100; Dako, Glostrup, Denmark), anti-CTTN (1∶200; Abcam, Cambridge, MA, USA) and anti-ERBB2 (1∶800; Cell Signaling Technology, Berverly, MA, USA) antibodies.

### Statistical analysis

Paired t test and Fisher's exact test were used. Differences at *P*<0.05 were considered statistically significant.

## Results

### Genomic clonality and heterogeneity in mucosal and submucosal portions of SMGC

To investigate the involvement of genomic CNAs in the process of submucosal invasion, we first compared the number of CNAs between paired MU and SM samples from the 23 SMGCs (Figure 2A). Eleven of the 23 cases showed an increased number of CNAs in the SM portion as compared with the MU portion, 11 showed a decreased number, and the remaining one case showed no change (Figure 2A). As a result, there was no statistically significant difference in the number of CNAs between paired MU and SM portions (Figure 2A, not significant in paired t-test). Furthermore, to identify CNAs specifically associated with submucosal invasion, we compared the averaged frequencies of CNAs in the MU portion with those in the paired SM portion (Figure 2B), but were unable to find any.

To investigate the difference of CNAs between MU and SM from the same tumor, we compared the genomic profiles of paired MU and SM in each case. One representative case is shown in Figure 2C, D, E and F. The paired MU and SM samples shared a similar pattern of genomic aberration in chromosome 9p (Figure 2D). However, there were distinct genomic aberrations in chromosomes 7p and 11 in the same case, as shown in Figure 2E and F. Amplification of 7p12 was observed only in MU, but not in SM (Figure 2E), and gain of chromosome 11 was observed only in SM, but not in MU (Figure 2F). These results suggested that tumor cells in the MU and SM of this case were clonally related, but composed of genetically heterogeneous subpopulations.

**Figure 3:**
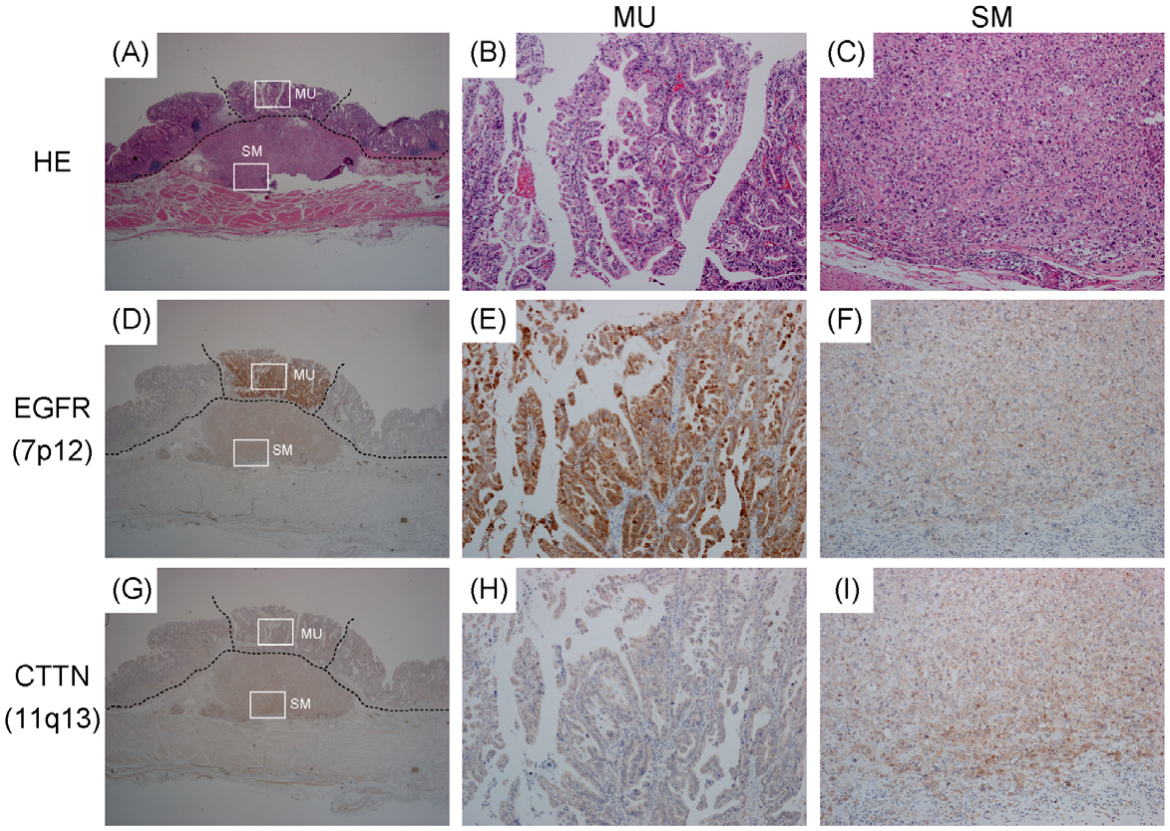
Immunohistochemical analysis of EGFR and CTTN expression pattern in Case 4. HE staining (A–C), and immunohistochemistry with antibodies against EGFR (D–F) and CTTN (G–I) are shown in low- (A, D and G) and high- (B, C, E, F, H and I) power views. EGFR, which was amplified only in the MU portion (see Figure 2E), is strongly positive only in the MU portion (D, E and F). Meanwhile, expression of CTTN, which was gained only in SM (see Figure 2F), shows higher positivity in SM than in MU (G, H and I).

Next, to determine whether the tumor cells showing amplification of 7p12 and those showing gain of 11q13 of case 4 were really limited to the MU and SM, respectively, we analyzed tissue sections from case 4 by immunohistochemistry with antibodies against EGFR, which was amplified only in the MU portion (Figure 2E), and CTTN, which was gained only in the SM portion (Figure 2F). As shown in Figure 3, positive immunoreactivity for EGFR was limited to the MU portion (Figure 3D, E and F), whereas only the SM portion showed strong immunoreactivity for CTTN (Figure 3G, H and I). These results suggested that, in case 4, the tumor cells with 7p amplification in MU could not have invaded the SM, whereas those with chromosome 11 gain might have invaded the SM.

Next, we analyzed genomic clonality and heterogeneity in the MU and SM of other cases. Among the other 22 cases, 14 showed a similar pattern of genomic aberration in the MU and SM (Figures S1 (6 cases) and S2 (8 cases)), suggesting that the cancer cells in the MU and SM of these cases were clonally related. Interestingly, 12 of the 14 cases showed a significant difference in the genomic profile patterns between MU and SM (Figures S1 (6 cases) and S2 (6 cases)), suggesting that these cases were also composed of genetically heterogeneous subpopulations.

### Genomic clonality and heterogeneity in primary (SM) and metastatic (LN) portions of SMGC

**Figure 4:**
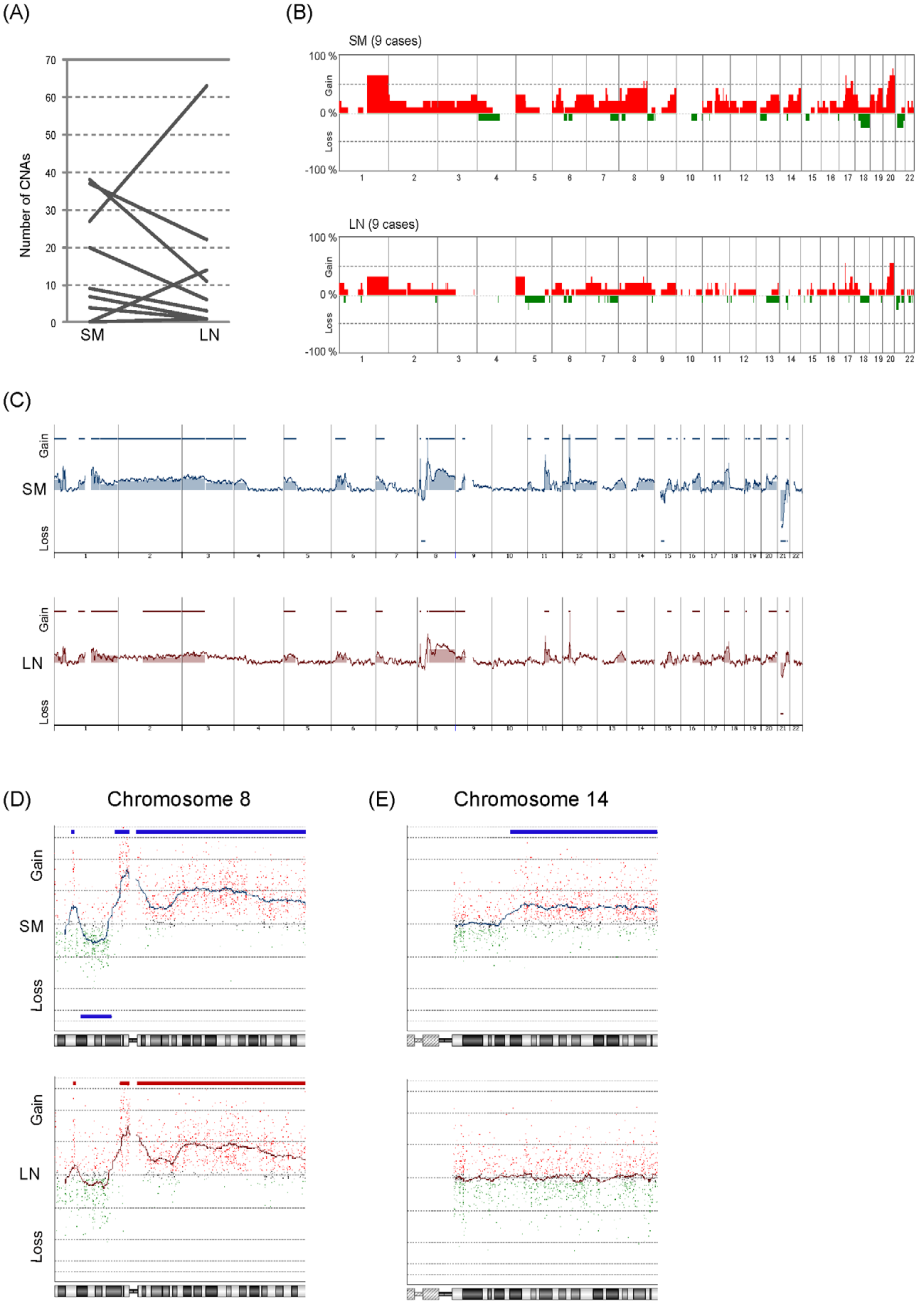
Comparison of CNAs between the paired SM and LN portions. (A) Comparison of the number of CNAs in the SM and LN portions. For this analysis, samples indicated by ‘c’ and ‘d’ in Table 1 were used. (B) Genome-wide frequencies of CNAs in the SM and corresponding paired LN in 9 cases. Horizontal lines: oligonucleotide probes are shown in order from chromosomes 1 to 22. Within each chromosome, clones are shown in order from the p telomere to the q telomere. Vertical lines: frequency (%) of gains (positive axis) and losses (negative axis) are shown for each probe. (C, D and E) Representative genomic profile of the SM and LN portions of SMGC. Whole genomic profiles of paired SM (above) and LN (below) portions from case 9 are shown in (C). Detailed genomic profiles of Chr8 and Chr14 are shown in (D) and (E), respectively. Horizontal lines above the center represent regions of gain, and those below the center represent regions of loss. Both SM and LN show similar genomic patterns in chromosome 8 (D). However, gain of chromosome 14q is detected only in the SM portion (E).

Next, to investigate the involvement of CNAs in the process of lymph node metastasis of early gastric cancer, we compared the number of CNAs between paired primary (SM) and metastatic (LN) portions of 9 SMGCs (Figure 4A). Three of the 9 cases showed an increased number of CNAs in the LN portion, whereas the remaining 6 cases showed a decrease (Figure 4A). As a result, there was no significant difference in the number of CNAs between the paired SM and LN portions (Figure 4A, not significant in paired t-test). Furthermore, to identify CNAs specifically associated with LN metastasis, we compared the averaged frequencies of CNAs in SM with those in the paired LN portion (Figure 4B), but were unable to find any.

To investigate the difference of CNAs between SM and LN of the same tumor, we compared the genomic profiles of paired SM and LN samples in each case. A representative case is shown in Figure 4C, D and E. The paired SM and LN samples shared a similar pattern of genomic aberration in chromosome 8 (Figure 4D), suggesting that both portions were derived from the same clonal origin. However, gain of chromosome 14 was observed only in SM, but not in LN (Figure 4E). These results suggested that the tumor cells in the SM and LN portions of this case were clonally related, but composed of genetically heterogeneous subpopulations.

We also analyzed genomic clonality and heterogeneity in SM and LN portions from other cases. Among the other 8 cases, 5 showed a similar pattern of genomic aberration in both SM and LN (Figure S3), suggesting that the paired SM and LN portions from these cases were clonally related. Furthermore, 4 of the 5 cases showed a significant difference in the genomic profile patterns between SM and LN (Figure S3), suggesting that these cases were also composed of genetically heterogeneous subpopulations.

### Comparison of genomic profiles between metastatic and non-metastatic SMGC

**Figure 5:**
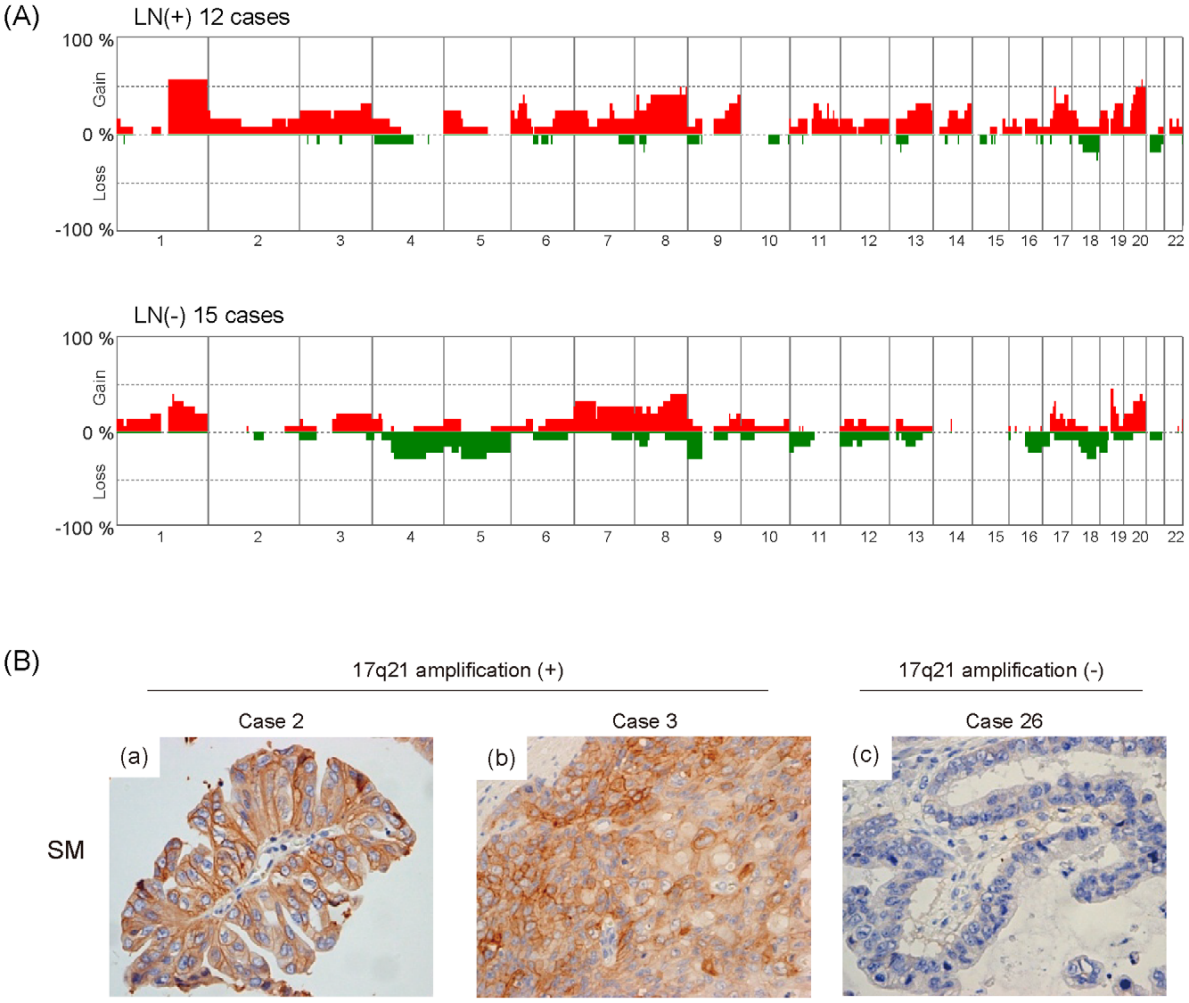
Comparison of CNAs between SMGC with and without lymph node metastasis. (A) Frequency (%) of gains (positive axis) and losses (negative axis) in 12 SMGCs with lymph node metastasis (LN(+) 12 cases) and 15 SMGCs without lymph node metastasis (LN(−) 15 cases) are shown. For this analysis, samples indicated by ‘e’ and ‘f’ in Table 1 were used. (B) Immunohistochemistry with anti-ERBB2 antibody. Primary SM (a, b and c) portions are immunostained with the antibody against ERBB2. Cases with amplification at 17q21 showed strong immunoreactivity for ERBB2 (a and b), while cases without such amplification did not (c).

Since no statistically significant differences were detected in the frequencies of CNAs between paired SM and LN portions (Figure 4B), we hypothesized that subpopulations carrying metastasis-related CNAs might be present in the SM as well as the LN portion of metastatic SMGC. Therefore, we next compared the frequencies of CNAs in the SM portion of metastatic SMGCs (12 cases) with those of non-metastatic SMGCs (15 cases), and found that gains at 11q13, 11q14, 11q22 and 14q32 were detected more frequently in metastatic SMGCs than in non-metastatic SMGCs (Figure 5A and Table 2). We also compared the frequencies of high-level copy number aberrations, such as amplification and deletion, between the two groups, and found that amplification of 17q21 was detected more frequently in metastatic SMGCs than in non-metastatic SMGCs (Table 3 and Table S1). These results suggested that gains at 11q13, 11q14, 11q22, 14q32 and amplification at 17q21 are involved in the LN metastasis of SMGCs.

**Table 2:** Comparison of CNAs between metastatic and non-metastatic SMGC.

Chromosomal band	Chromosomal region (bp)	meta(+) n = 12	meta(−) n = 15	Fisher's exact test *p* value
**Gains**				
11q13.1–q13.5	66189604–76676099	4	0	0.028
11q14.1	76713358–78303305	4	0	0.028
11q22.2–q22.3	101630495–102844567	4	0	0.028
14q32.2–q32.33	98389742–105000952	4	0	0.028
**Losses**				
none				

**Table 3:** Minimal common regions of amplifications and deletions in SMGCs.

Choromosomal band	Chromosomal region (bp)	meta(+) n = 12	meta(−) n = 15	Fisher's exact test p value	Candidate gene
**Amplification**					
8p23.1	10811845–11770357	2	0	0.19	XKR6, MTMR9, C8orf13, BLK, GATA4, NEIL2, FDFT1, CTSB
14q22.1	50171710–50181479	0	2	0.49	SAV1
17q21	35076296–35139027	4	0	0.028	TCAP,PNMT,PERLD1,ERBB2,C17orf37
19q12	34978732–35127210	0	5	0.047	CCNE1,C19orf2
**Deletion**					
none					

Detailed information regarding the size of regions in each case is shown in Table S1.

The minimal common region of amplification at 17q21 contained 5 genes listed in Table 3. Since ERBB2, a well known oncogene [26], [27], [28], was included in the list, we carried out immunohistochemical analysis of ERBB2 overexpression in all 27 cases. As shown in Figure 5B, cases with 17q21 amplification exhibited strong staining for ERBB2 in SM, whereas one case without amplification did not. Furthermore, ERBB2 overexpression was significantly associated with 17q21 amplification (Table 4), suggesting that ERBB2 amplification and overexpression may be involved in the LN metastasis of a proportion of SMGCs.

**Table 4:** Relationship between ERBB2 amplification and overexpression.

17q21 amplification	ERBB2 overexpression (immunohistochemistry)		total
	positive cases (%)	negative cases (%)	
+	4 (100%)	0 (0%)	4
−	0 (0%)	23 (100%)	23
total	4	23	27

## Discussion

**Figure 6:**
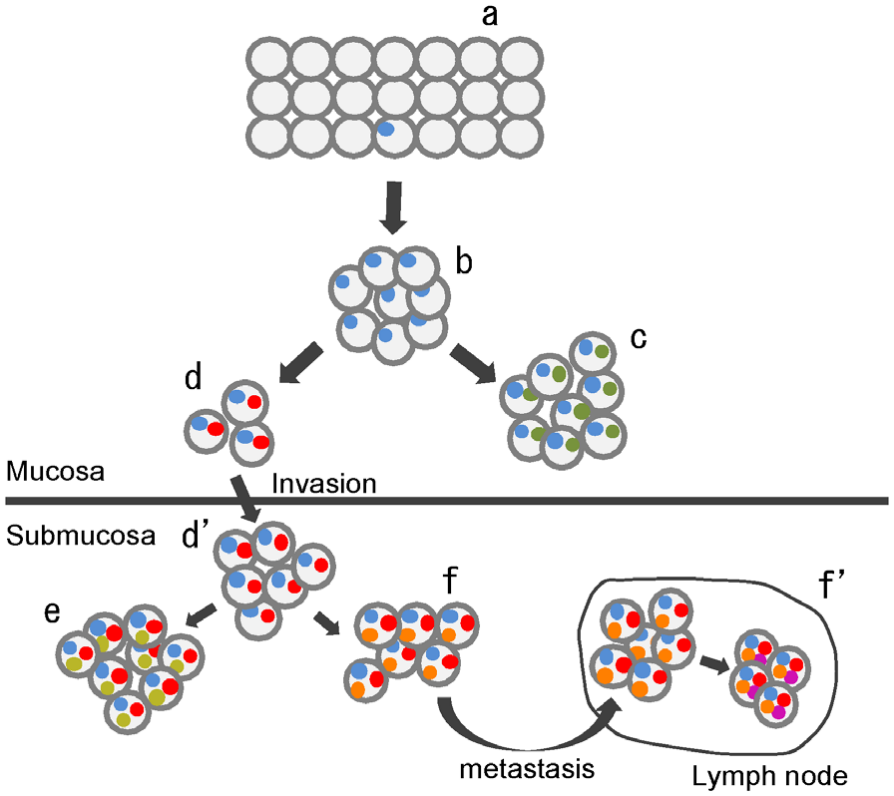
Hypothetical model for the submucosal invasion and lymph node metastasis in early gastric cancer. The horizontal line in the center of the figure indicates the muscularis mucosa. Gray circles indicate tumor cells. Colored small circles indicate genomic aberrations. Gastric tumors arise from a single cell with one (or few) genomic aberration (a). The single clone then proliferates more effectively than its neighbors (b). During the process of proliferation in the gastric mucosa, some tumor cells acquire new mutations at random. Subsequently, each of genetically distinct subclones forms a unique subpopulation (c and d). Among these subpopulations, only one(s) with the capacity for invasion can pass through the muscularis mucosa and proliferate in the submucosa (d and d′). Importantly, other clones cannot invade into the submucosa (c), but can proliferate and form subpopulations genetically distinct from the invasive one. After invasion, one (or a few) subpopulation again develops further genetically distinct subpopulations through clonal evolution (e and f), and one with the capacity for metastasis can spread to lymph nodes (f and f′). Thus, the primary tumor mass becomes heterogeneous as a consequence of clonal evolution.

It is widely accepted that a tumor arises from a single cell. However, how it progresses to an advanced stage is still being debated. Early studies of colorectal and pancreatic cancers led to a notion that the development and progression of these cancers are associated with accumulation of chromosomal aberrations, referred to as the multistep tumorigenesis model [29], [30]. For example, genomic aberrations of the APC, KRAS, SMAD4 and TP53 genes are involved in the adenoma-carcinoma sequence in the colon [29]. However, such studies focused on only a proportion of tumor-related genes, and neglected the role of most other genes. Furthermore, this model was unable to evaluate the significance of intratumoral genomic heterogeneity for tumor development and progression. Meanwhile, recent studies have led to the establishment of another model, designated the clonal evolution model [7], [9], [10]. In this model, a single clone evolves into several distinct subpopulations through the accumulation of diverse genetic abnormalities. The predominant population may be replaced by distinct subpopulations within a single tumor mass through the effects of environmental selection pressure and/or the stage of tumor progression. As a consequence, several genetically heterogeneous cell populations may coexist within a single tumor mass. Evidence of intratumoral genetic heterogeneity associated with clonal evolution has been obtained for a variety of solid tumors, including prostate cancer [14], Barrett's esophagus [31], ovarian cancer [32], [33], cervical cancer [34], breast cancer [15], [35], neuroblastoma [36], pancreatic cancer [13], [37], and colorectal cancer [38]. Interestingly, in a study of lethal metastatic prostate cancer, no CNAs specifically related to the site of metastasis were found [14]. Similarly, in a study of high-grade serous ovarian carcinoma, there was no evidence for a relationship between acquisition of cisplatin resistance and specific CNAs [39]. These results suggest that the multistep tumorigenesis model, in which specific aberrations play important roles in tumor development and progression, does not always represent the way in which tumors acquire their malignant character. In the present study, we initially hypothesized that acquisition of specific CNA(s) might be important for submucosal invasion. However, we were unable to find any CNAs that were more frequent in SM than in the paired MU sample. Furthermore, we also observed no significant difference regarding the number of CNAs in the paired MU and SM portions. However, we found that the majority of SMGCs were composed of clonally-related, but genetically distinct subpopulations, suggesting that clonal evolution may occur during the progression of gastric cancer. Taken together, although the number of cases examined was limited, our findings suggested that generation of genetically different subpopulations rather than acquisition of specific CNAs in the MU portion may be important for the process of submucosal invasion. On the basis of these findings, we propose a hypothetical model for the process of SM invasion and LN metastasis of early gastric cancer (Figure 6). To confirm this hypothesis, further studies with larger samples will be required.

Our data indicating that SMGCs are composed of genetically heterogeneous subpopulations are important in the context of gastric cancer research and treatment, because tumor heterogeneity makes the development of effective drugs difficult. Since genomic CNAs have an impact on gene expression profiles in various cancers [16], [21], [40], [41], [42], [43], it is possible that each of the genetically distinct subpopulations within a single tumor may differ in both biological behavior and response to anticancer drugs, including molecular targeting agents. Cooke et al. have proposed that clarification of different genetic subpopulations within a single tumor would allow effective therapy employing a specific agent targeting a common genomic aberration or combined agents targeting unique genomic aberrations in each of the distinct subpopulations [39]. This strategy may also applicable to the treatment of gastric cancer.

Among the 23 cases we analyzed, 15 showed a clonal relationship between the MU and SM portions. Furthermore, 13 of the latter 15 cases also showed differences in CNAs between the two regions, suggesting that clonal evolution frequently occurs in the early phase of gastric carcinogenesis. The relationship between the paired MU and SM samples in the other 8 cases without common CNAs remained unclear. Two possible explanations for this can be suggested. One is that tumors in the paired portions, which did not have common CNAs, developed independently. The other is that the paired portions shared other types of genetic aberrations, such as mutations and translocations, which cannot be detected by array CGH. In the latter case, next-generation sequencing might be useful for analyzing such relationships.

In this study, gains at 11q13, 11q14, 11q22, and 14q32, and amplification at 17q21, were more frequent in the SM portion of metastatic SMGCs than in those of non-metastatic SMGCs. Interestingly, gains at 11q13 and 14q32 are reportedly involved in liver metastasis of colon cancer [38]. Therefore, these data suggest that gain at 11q13 and 14q32 may be involved in the metastasis of gastrointestinal cancers. Chromosome 17q21 harbors a potent oncogene, ERBB2. Association of ERBB2 expression with the clinicopathological features of gastric cancer has been investigated in several studies [44], [45], [46], [47], [48], [49]. However, the influence of ERBB2 overexpression on LN metastasis differed among those studies [44], [46], [47]. In the present study, despite the limited number of SMGCs examined, all of those with ERBB2 amplification and overexpression showed lymph node metastasis. Further study using a larger number of SMGCs will be required to evaluate the significance of this tendency.

## Supporting Information

Figure S1 **Cases showing both common and different genomic aberrations between the MU and SM portions.** The left panels show common patterns of genomic aberrations in MU and SM for each case. The center and right panels show different patterns of genomic aberration between the two portions in each case.
(TIF)

Figure S2 **Cases showing both common and different genomic aberrations between the MU and SM portions.** Common and different patterns of genomic aberration between MU and SM for each case are shown.
(TIF)

Figure S3 **Cases showing both common and different genomic aberrations between the SM and LN portions.** The left panels show common patterns of genomic aberration between SM and LN for each case. The center and right panels show different patterns of genomic aberration between the two portions in each case.
(TIF)

Table S1 **Recurrent amplifications and deletions in SMGCs.**
(DOC)

## References

[1] Parkin DM, Bray F, Ferlay J, Pisani P (2005). Global cancer statistics, 2002.. CA Cancer J Clin.

[2] Haruta H, Hosoya Y, Sakuma K, Shibusawa H, Satoh K (2008). Clinicopathological study of lymph-node metastasis in 1,389 patients with early gastric cancer: assessment of indications for endoscopic resection.. J Dig Dis.

[3] Kwee RM, Kwee TC (2008). Predicting lymph node status in early gastric cancer.. Gastric Cancer.

[4] Vogelstein B, Kinzler KW (2004). Cancer genes and the pathways they control.. Nat Med.

[5] Ried T, Heselmeyer-Haddad K, Blegen H, Schrock E, Auer G (1999). Genomic changes defining the genesis, progression, and malignancy potential in solid human tumors: a phenotype/genotype correlation.. Genes Chromosomes Cancer.

[6] Uchida M, Tsukamoto Y, Uchida T, Ishikawa Y, Nagai T (2010). Genomic profiling of gastric carcinoma in situ and adenomas by array-based comparative genomic hybridization.. J Pathol.

[7] Merlo LM, Pepper JW, Reid BJ, Maley CC (2006). Cancer as an evolutionary and ecological process.. Nat Rev Cancer.

[8] Heng HH, Bremer SW, Stevens JB, Ye KJ, Liu G (2009). Genetic and epigenetic heterogeneity in cancer: a genome-centric perspective.. J Cell Physiol.

[9] Heng HH, Stevens JB, Bremer SW, Ye KJ, Liu G (2010). The evolutionary mechanism of cancer.. J Cell Biochem.

[10] Polyak K (2008). Is breast tumor progression really linear?. Clin Cancer Res.

[11] Davies JJ, Wilson IM, Lam WL (2005). Array CGH technologies and their applications to cancer genomes.. Chromosome Res.

[12] Harada K, Nishizaki T, Ozaki S, Kubota H, Ito H (1998). Intratumoral cytogenetic heterogeneity detected by comparative genomic hybridization and laser scanning cytometry in human gliomas.. Cancer Res.

[13] Harada T, Okita K, Shiraishi K, Kusano N, Kondoh S (2002). Interglandular cytogenetic heterogeneity detected by comparative genomic hybridization in pancreatic cancer.. Cancer Res.

[14] Liu W, Laitinen S, Khan S, Vihinen M, Kowalski J (2009). Copy number analysis indicates monoclonal origin of lethal metastatic prostate cancer.. Nat Med.

[15] Torres L, Ribeiro FR, Pandis N, Andersen JA, Heim S (2007). Intratumor genomic heterogeneity in breast cancer with clonal divergence between primary carcinomas and lymph node metastases.. Breast Cancer Res Treat.

[16] Gorringe KL, Boussioutas A, Bowtell DD (2005). Novel regions of chromosomal amplification at 6p21, 5p13, and 12q14 in gastric cancer identified by array comparative genomic hybridization.. Genes Chromosomes Cancer.

[17] Nakayama T, Ling ZQ, Mukaisho K, Hattori T, Sugihara H (2010). Lineage analysis of early and advanced tubular adenocarcinomas of the stomach: continuous or discontinuous?. BMC Cancer.

[18] Peng DF, Sugihara H, Mukaisho K, Ling ZQ, Hattori T (2004). Genetic lineage of poorly differentiated gastric carcinoma with a tubular component analysed by comparative genomic hybridization.. J Pathol.

[19] Takada H, Imoto I, Tsuda H, Sonoda I, Ichikura T (2005). Screening of DNA copy-number aberrations in gastric cancer cell lines by array-based comparative genomic hybridization.. Cancer Sci.

[20] Tomioka N, Morita K, Kobayashi N, Tada M, Itoh T (2010). Array comparative genomic hybridization analysis revealed four genomic prognostic biomarkers for primary gastric cancers.. Cancer Genet Cytogenet.

[21] Tsukamoto Y, Uchida T, Karnan S, Noguchi T, Nguyen LT (2008). Genome-wide analysis of DNA copy number alterations and gene expression in gastric cancer.. J Pathol.

[22] Vauhkonen H, Vauhkonen M, Sajantila A, Sipponen P, Knuutila S (2006). DNA copy number aberrations in intestinal-type gastric cancer revealed by array-based comparative genomic hybridization.. Cancer Genet Cytogenet.

[23] Vauhkonen H, Vauhkonen M, Sipponen P, Knuutila S (2007). Oligonucleotide array comparative genomic hybridization refines the structure of 8p23.1, 17q12 and 20q13.2 amplifications in gastric carcinomas.. Cytogenet Genome Res.

[24] Weiss MM, Kuipers EJ, Postma C, Snijders AM, Pinkel D (2004). Genomic alterations in primary gastric adenocarcinomas correlate with clinicopathological characteristics and survival.. Cell Oncol.

[25] Yang S, Jeung HC, Jeong HJ, Choi YH, Kim JE (2007). Identification of genes with correlated patterns of variations in DNA copy number and gene expression level in gastric cancer.. Genomics.

[26] Bargmann CI, Hung MC, Weinberg RA (1986). The neu oncogene encodes an epidermal growth factor receptor-related protein.. Nature.

[27] Di Fiore PP, Pierce JH, Kraus MH, Segatto O, King CR (1987). erbB-2 is a potent oncogene when overexpressed in NIH/3T3 cells.. Science.

[28] Semba K, Kamata N, Toyoshima K, Yamamoto T (1985). A v-erbB-related protooncogene, c-erbB-2, is distinct from the c-erbB-1/epidermal growth factor-receptor gene and is amplified in a human salivary gland adenocarcinoma.. Proc Natl Acad Sci U S A.

[29] Vogelstein B, Fearon ER, Hamilton SR, Kern SE, Preisinger AC (1988). Genetic alterations during colorectal-tumor development.. N Engl J Med.

[30] Wilentz RE, Iacobuzio-Donahue CA, Argani P, McCarthy DM, Parsons JL (2000). Loss of expression of Dpc4 in pancreatic intraepithelial neoplasia: evidence that DPC4 inactivation occurs late in neoplastic progression.. Cancer Res.

[31] Maley CC, Galipeau PC, Finley JC, Wongsurawat VJ, Li X (2006). Genetic clonal diversity predicts progression to esophageal adenocarcinoma.. Nat Genet.

[32] Khalique L, Ayhan A, Weale ME, Jacobs IJ, Ramus SJ (2007). Genetic intra-tumour heterogeneity in epithelial ovarian cancer and its implications for molecular diagnosis of tumours.. J Pathol.

[33] Cooke SL, Ng CK, Melnyk N, Garcia MJ, Hardcastle T Genomic analysis of genetic heterogeneity and evolution in high-grade serous ovarian carcinoma.. Oncogene.

[34] Cooke SL, Temple J, Macarthur S, Zahra MA, Tan LT Intra-tumour genetic heterogeneity and poor chemoradiotherapy response in cervical cancer.. Br J Cancer.

[35] Aubele M, Mattis A, Zitzelsberger H, Walch A, Kremer M (1999). Intratumoral heterogeneity in breast carcinoma revealed by laser-microdissection and comparative genomic hybridization.. Cancer Genet Cytogenet.

[36] Mora J, Cheung NK, Gerald WL (2001). Genetic heterogeneity and clonal evolution in neuroblastoma.. Br J Cancer.

[37] Yachida S, Jones S, Bozic I, Antal T, Leary R (2010). Distant metastasis occurs late during the genetic evolution of pancreatic cancer.. Nature.

[38] Sayagues JM, Abad Mdel M, Melchor HB, Gutierrez ML, Gonzalez-Gonzalez M (2010). Intratumoural cytogenetic heterogeneity of sporadic colorectal carcinomas suggests several pathways to liver metastasis.. J Pathol.

[39] Cooke SL, Ng CK, Melnyk N, Garcia MJ, Hardcastle T (2010). Genomic analysis of genetic heterogeneity and evolution in high-grade serous ovarian carcinoma.. Oncogene.

[40] Heidenblad M, Lindgren D, Veltman JA, Jonson T, Mahlamaki EH (2005). Microarray analyses reveal strong influence of DNA copy number alterations on the transcriptional patterns in pancreatic cancer: implications for the interpretation of genomic amplifications.. Oncogene.

[41] Hyman E, Kauraniemi P, Hautaniemi S, Wolf M, Mousses S (2002). Impact of DNA amplification on gene expression patterns in breast cancer.. Cancer Res.

[42] Jarvinen AK, Autio R, Haapa-Paananen S, Wolf M, Saarela M (2006). Identification of target genes in laryngeal squamous cell carcinoma by high-resolution copy number and gene expression microarray analyses.. Oncogene.

[43] Yoshimoto T, Matsuura K, Karnan S, Tagawa H, Nakada C (2007). High-resolution analysis of DNA copy number alterations and gene expression in renal clear cell carcinoma.. J Pathol.

[44] Barros-Silva JD, Leitao D, Afonso L, Vieira J, Dinis-Ribeiro M (2009). Association of ERBB2 gene status with histopathological parameters and disease-specific survival in gastric carcinoma patients.. Br J Cancer.

[45] Falck VG, Gullick WJ (1989). c-erbB-2 oncogene product staining in gastric adenocarcinoma. An immunohistochemical study.. J Pathol.

[46] Kim MA, Jung EJ, Lee HS, Lee HE, Jeon YK (2007). Evaluation of HER-2 gene status in gastric carcinoma using immunohistochemistry, fluorescence in situ hybridization, and real-time quantitative polymerase chain reaction.. Hum Pathol.

[47] Park DI, Yun JW, Park JH, Oh SJ, Kim HJ (2006). HER-2/neu amplification is an independent prognostic factor in gastric cancer.. Dig Dis Sci.

[48] Park JB, Rhim JS, Park SC, Kimm SW, Kraus MH (1989). Amplification, overexpression, and rearrangement of the erbB-2 protooncogene in primary human stomach carcinomas.. Cancer Res.

[49] Takehana T, Kunitomo K, Kono K, Kitahara F, Iizuka H (2002). Status of c-erbB-2 in gastric adenocarcinoma: a comparative study of immunohistochemistry, fluorescence in situ hybridization and enzyme-linked immuno-sorbent assay.. Int J Cancer.

