# Supplementary figures and images for: Genomic Profiling of Submucosal-Invasive Gastric Cancer by Array-Based Comparative Genomic Hybridization
Source: PLoS One. 2011 Jul 21;6(7):e22313. doi: 10.1371/journal.pone.0022313 (PMC3141024; doi:10.1371/journal.pone.0022313)

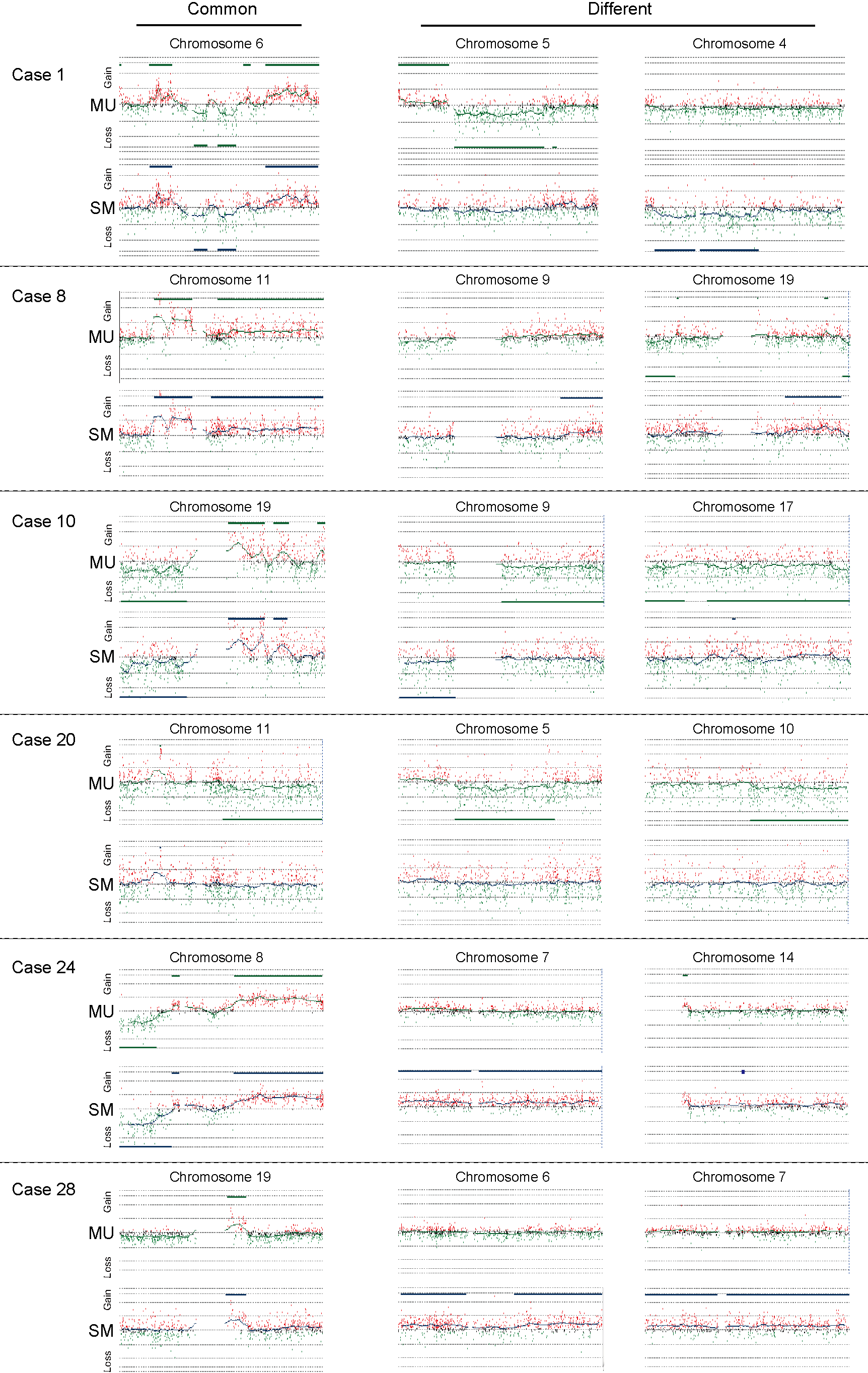

Supplement: Figure S1 — Cases showing both common and different genomic aberrations between the MU and SM portions. The left panels show common patterns of genomic aberrations in MU and SM for each case. The center and right panels show different patterns of genomic aberration between the two portions in each case. (TIF) [file pone.0022313.s001.tif]

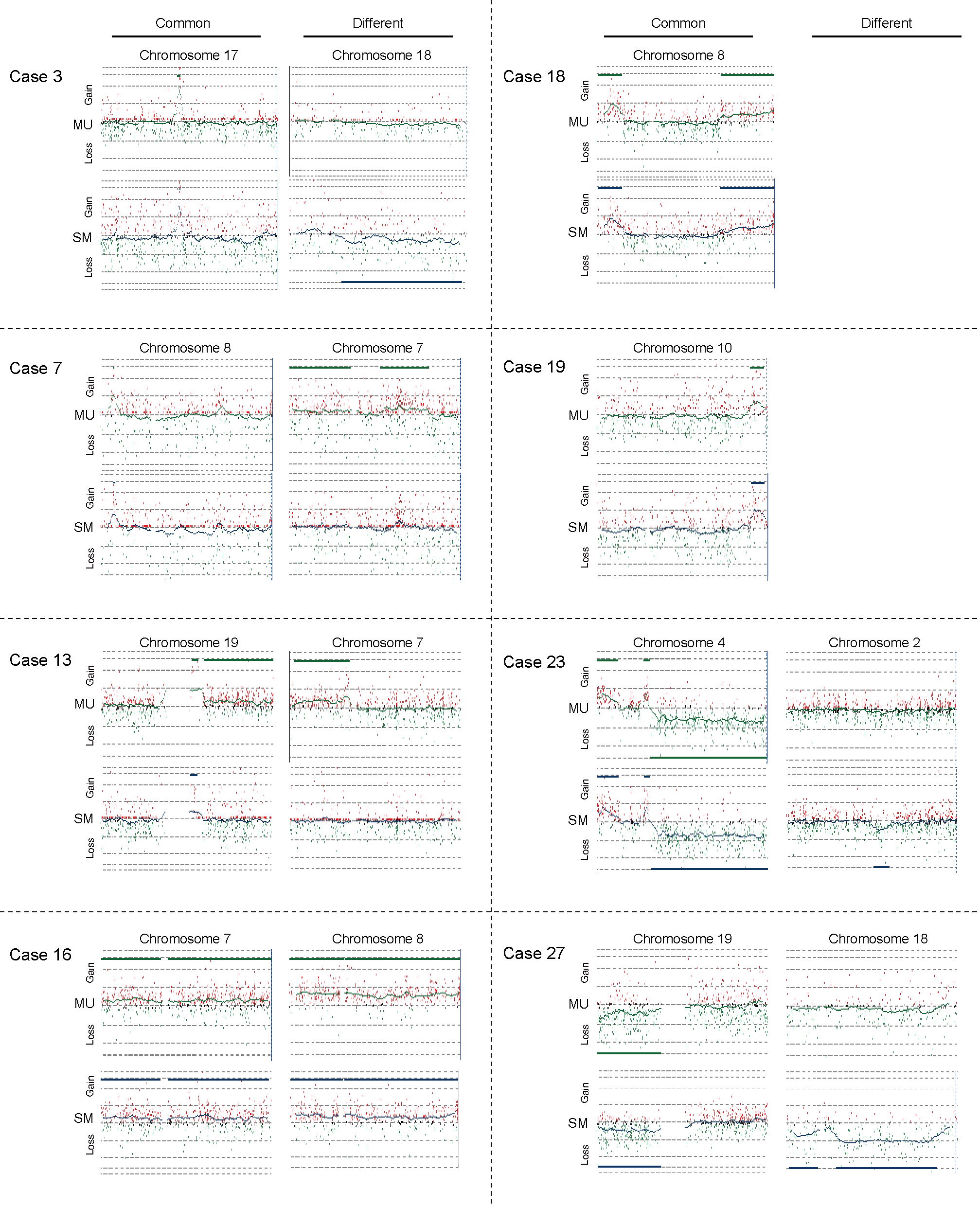

Supplement: Figure S2 — Cases showing both common and different genomic aberrations between the MU and SM portions. Common and different patterns of genomic aberration between MU and SM for each case are shown. (TIF) [file pone.0022313.s002.tif]

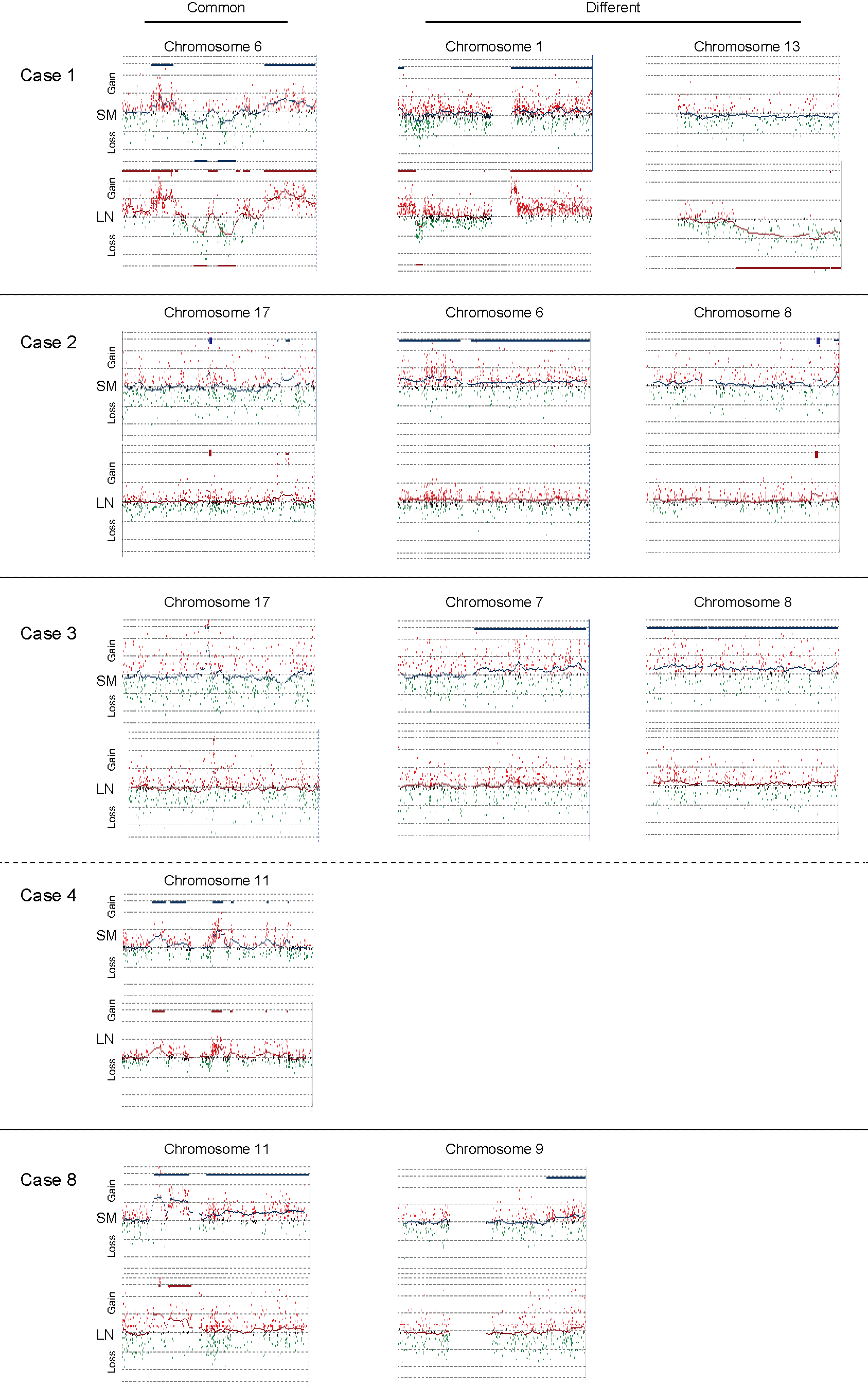

Supplement: Figure S3 — Cases showing both common and different genomic aberrations between the SM and LN portions. The left panels show common patterns of genomic aberration between SM and LN for each case. The center and right panels show different patterns of genomic aberration between the two portions in each case. (TIF) [file pone.0022313.s003.tif]
