# Supplementary material for: Genomic Profiling of Submucosal-Invasive Gastric Cancer by Array-Based Comparative Genomic Hybridization
Source: PLoS One. 2011 Jul 21;6(7):e22313. doi: 10.1371/journal.pone.0022313 (PMC3141024; doi:10.1371/journal.pone.0022313)
Supplement: Table S1 — Recurrent amplifications and deletions in SMGCs. (DOC) [file pone.0022313.s004.doc]

| Choromosomal band |  | meta(+)  n=12 | meta(-)  n=15 |  | Chromosomal region (bp) | |  | Size (bp) |  | Genes |
| --- | --- | --- | --- | --- | --- | --- | --- | --- | --- | --- |
|  |  |  |  |  | Start | Stop |  |  |  |  |
| **Amplification** |  |  |  |  |  |  |  |  |  |  |
| 8p23.1 |  | Case 7 | - |  | 10323367 | 12711879 |  | 2388512 |  | MSRA, RP1L1, SOX7, PINX1, XKR6, MTMR9, C8orf13, BLK, GATA4, NEIL2, FDFT1, CTSB, LONRF1 |
|  |  | Case 9 | - |  | 10811845 | 11770357 |  | 958512 |  | XKR6, MTMR9, C8orf13, BLK, GATA4, NEIL2, FDFT1, CTSB |
| 14q22.1 |  | - | Case 20 |  | 50171710 | 50181479 |  | 9769 |  | SAV1 |
|  |  | - | Case 22 |  | 50171710 | 50181479 |  | 9769 |  | SAV1 |
| 17q21 |  | Case 1 | - |  | 33949805 | 35559815 |  | 1610010 |  | MLLT6, PCGF2, PSMB3, PIP4K2B, CCDC49, RPL23, LASP1, FBXO47, PLXDC1, CACNB1, RPL19, FBXL20, MED1, CRKRS, NEUROD2, PPP1R1B, STARD3, TCAP, PNMT, PERLD1, ERBB2, C17orf37, GRB7, IKZF3, ZPBP2, GSDML, ORMDL3, GSDM1, PSMD3, CSF3, MED24, THRA, NR1D1, CASC3 |
|  |  | Case 2 | - |  | 35076296 | 35221880 |  | 145584 |  | TCAP, PNMT, PERLD1, ERBB2, C17orf37, GRB7, IKZF3 |
|  |  | Case 3 | - |  | 34961387 | 35506166 |  | 544779 |  | NEUROD2, PPP1R1B, STARD3, TCAP, PNMT, PERLD1, ERBB2, C17orf37, GRB7, IKZF3, ZPBP2, GSDML, ORMDL3, GSDM1, PSMD3, CSF3, MED24, THRA, NR1D1 |
|  |  | Case 8 | - |  | 34925507 | 35139027 |  | 213520 |  | NEUROD2, PPP1R1B, STARD3, TCAP, PNMT, PERLD1, ERBB2, C17orf37 |
| 19q12 |  | - | Case 10 |  | 34978732 | 35707776 |  | 729044 |  | CCNE1, C19orf2, ZNF536 |
|  |  | - | Case 13 |  | 33399431 | 36074781 |  | 2675350 |  | UQCRFS1, POP4, PLEKHF1, C19orf12, CCNE1, C19orf2, ZNF536 |
|  |  | - | Case 18 |  | 33198460 | 35707776 |  | 2509316 |  | UQCRFS1, POP4, PLEKHF1, C19orf12, CCNE1, C19orf2, ZNF536 |
|  |  | - | Case 20 |  | 33198460 | 36690656 |  | 3492196 |  | UQCRFS1, POP4, PLEKHF1, C19orf12, CCNE1, C19orf2, ZNF536, TSHZ3 |
|  |  | - | Case 27 |  | 34865030 | 35127210 |  | 262180 |  | UQCRFS1, POP4, PLEKHF1, C19orf12, CCNE1, C19orf2, ZNF536, TSHZ3 |
| **Deletion** |  |  |  |  |  |  |  |  |  |  |
| none |  | - | - |  |  |  |  |  |  |  |

**Table S1. Recurrent amplifications and deletions in SMGCs.**
